# A roadmap towards Ireland’s membership of BBMRI-ERIC

**DOI:** 10.12688/hrbopenres.14089.1

**Published:** 2025-04-04

**Authors:** Amina Arar, Heidi Annuk, Sean O. Hynes, Emma Snapes, Michael Kerin, Sonja Khan, Nicola Miller

**Affiliations:** 1 Institute for Clinical Trials, School of Medicine, University of Galway, Galway, Ireland; 2 Lambe Institute for Translational Research, School of Medicine, University of Galway, Galway, Ireland; 3 Discipline of Surgery, Lambe Institute for Translational Research, University of Galway, Galway, Ireland; 4 Division of Anatomical Pathology, Galway University Hospital, Galway, Ireland; 5 Discipline of Pathology, School of Medicine, University of Galway, Galway, Ireland; 6 BioConsulting, Cork, Ireland; 7 HRB Clinical Research Facility, School of Medicine, University of Galway, Galway, Ireland

**Keywords:** Biobanking, BBMRI-ERIC, Clinical Trials, European Research Infrastructure, Biobanking Standardisation, Healthcare Research Integration, Medical Research Networks, Transnational Collaboration

## Abstract

**Background:**

BioBANC Symposium has become a key forum for the Irish biobanking community. The third annual event focused on networking and quality, featuring a workshop to explore the role and value of networked biobanking for Ireland. The Director General of BBMRI-ERIC advanced these discussions by addressing an audience of interdisciplinary experts, high-level stakeholders, and policymakers from the Irish biobanking community, as well as representatives from the Health Research Charities Ireland (HRCI), the National Clinical Trials Office (NCTO), the European Clinical Research Infrastructure Network (ECRIN-ERIC) research infrastructure, and the Department of Health.

**Methods:**

A panel discussion brought together an array of experts from patient advocacy, clinical trials, pharmaceutical industry, government, healthcare policy and BBMRI-ERIC representation to explore the integration of Ireland into the BBMRI-ERIC network. This diverse panel discussed strategies to enhance Ireland’s biobanking capabilities and leverage international collaborations. Themes explored included the benefits, gaps and changes needed if Ireland was to consider membership of BBMRI-ERIC. VEVOX live polling was used to gauge audience views and questions on posed topics, enabling interactive audience participation and capturing real-time feedback to enrich the discussion.

**Results:**

Substantial benefits that BBMRI-ERIC membership would bring for Ireland were highlighted, including enhanced infrastructure, standardised practices, and greater economic opportunities. Attendees also delved into how Ireland could address current gaps and align its biobanking operations with broader European standards. The discussion identified several critical themes and recommendations to address the need for funding, legislative support, education, public engagement, and strategic planning.

**Conclusion:**

This article aims to encapsulate the discussions and outcomes of BioBANC Symposium III, focusing on the strategic moves Ireland must consider harnessing the full potential of its biobanking community. It serves not just as a record of proceedings but as a guide for action, urging stakeholders at all levels to collaborate towards a unified goal.

## Abbreviations

BBMRI-ERIC – Biobanking and Biomolecular Resources Research Infrastructure - European Research Infrastructure Consortium

BnaG – BioBANC na Gaillimhe

CRF – Clinical Research Facility

DOH – Department of Health

EATRIS-ERIC – European Infrastructure for Translational Medicine - European Research Infrastructure Consortium

ECRIN – European Clinical Research Infrastructure Network

EHDS – European Health Data Space

ERIC – European Research Infrastructure Consortium

EU-AMRI – European Union Alliance for Medical Research Infrastructures

GDPR – General Data Protection Regulation

HRCI – Health Research Charities Ireland

HRB – Health Research Board

IARC – International Agency for Research on Cancer

INAB – Irish National Accreditation Board 

ISBER – International Society for Biological and Environmental Repositories

NBWG – National Biobanking Working Group

NCTO – National Clinical Trials Office

NREC – National Research Ethics Committees

NSAI – National Standards Authority of Ireland

PFM – Personalised Functional Medicine

PPI – Patient and Public Involvement

WHO – World Health Organisation

## Introduction

Biobanks are the cornerstone of biomedical research and healthcare delivery as medicine becomes increasingly personalised. For common complex diseases such as cancer, much of our insight into the causes and treatment of disease has come from analysis of clinical samples. The third annual BioBANC Symposium held at the University of Galway on 6th September 2024, brought together experts and advocates from the Irish biobanking, clinical research, healthcare, industry, and patient advocacy communities. Central to the discussion was Ireland’s membership of Biobanking and Biomolecular Resources Research Infrastructure - European Research Infrastructure Consortium (BBMRI-ERIC), the European Research Infrastructure dedicated to strengthening biomedical research by supporting networked and standardised biobanking practices and policies across Europe in the provision of structured access to quality aligned samples and data ^1^.

In a welcome address, Professor Michael Kerin, Chair of Surgery, University of Galway, Director of the HSE West and North West Cancer Network and Vice President of the Royal College of Surgeons in Ireland ardently championed the patient’s voice highlighting the challenges of delivering cancer care, recruiting participants for biobanking and clinical trials amid the constraints of the General Data Protection Regulation (GDPR) and the impracticalities of reconsent. He also commented on the importance of biobanking as a key enabler in personalised medicine and advances in cancer care. This prefaced a workshop “Pathway to Ireland joining BBMRI-ERIC” with keynote presentations by Professor Jens Habermann, Director General of BBMRI-ERIC and Dr Eva Ortega-Paino, Secretary General for Research, Ministry of Science, Innovation and Universities, Spain ( Table 1).

**Table 1.** Future of Irish Biobanking Community - Roadmap towards BBMRI-ERIC membership panellists.

Panellist / Title	Representation	Affiliation
Mr Billy McCann Member	Patient and Public Involvement (PPI)	National Research Ethics Committee (NREC) Medical Devices National Biobanking Working Group (NBWG)
Dr Avril Kennan CEO	Patient Advocacy	Health Research Charities Ireland (HRCI)
Prof Fai Ng Director	Clinical Trials	Clinical Research Facility (CRF) University College Cork (UCC) National Clinical Trials Office (NCTO)
Dr Robert O’Connor Director	Clinical Trials (ECRIN) Research Infrastructure	HRB-National Clinical Trials Office (NCTO), UCC
Ms Orlaith Gavan Country Medical Director	Pharmaceutical Industry	Pfizer Healthcare, Ireland
Dr Eva Ortega-Paino Secretary General	Government Representative	Ministry of Science, Innovation and Universities, Spain
Professor Jens Habermann Director General	European Research Infrastructure	BBMRI-ERIC

In an opening presentation titled “Setting the Scene of Biobanking in Ireland”, Oonagh Ward, Head of Research and Innovation Infrastructures, Health Research Board (HRB) outlined previous Irish biobanking position papers and why biobanking infrastructure in Ireland has failed to date. Reference was made to the HRB’s commitment to biobanking as a key action in the HRB strategy 2021–2025 to: “Take a leading role to convene stakeholders to progress the design, development and implementation of national, shared high-cost research infrastructures, including in the areas of biobanking and genomic research” ^2^. A vision towards BBMRI-ERIC membership was signposted using experience from the strategic investment in clinical trials in Ireland to standardise expertise and delivery through membership of the European Clinical Research Infrastructure Network (ECRIN) the European research infrastructure (ERIC) for clinical trials as precedence.

Professor Jens Habermann, Director General of BBMRI-ERIC, highlighted the extensive capabilities of the BBMRI-ERIC network in his keynote address, “Connecting Ireland to Europe's Biobanking and Biomolecular Resources Research Infrastructure.” As one of the largest distributed research infrastructures, with its headquarters in Graz, Austria, BBMRI-ERIC represents almost 500 biobanks across 25 Member and Observer countries, including the International Agency for Research on Cancer (IARC)/World Health Organisation (WHO). It offers scientific services for ethical, legal and societal issues, quality management, IT services, biobanking development, and provides central services and functions such as finance and project management, public affairs, and outreach, education and communications. It furthermore operates the world’s largest biobank catalogue – the BBMRI-ERIC Directory.

The scale of BBMRI-ERIC’s connectivity to a vast network of partners and EU initiatives illustrates its extensive reach. This network includes the ERIC Forum, which BBMRI-ERIC coordinates, and other European Research Infrastructures in the field of health and life sciences. With a coordinating role in projects relating to EU priorities such as EU Mission on Cancer or further strengthening of Research Infrastructures and ERICs, as well as an important role in other projects, BBMRI-ERIC is contributing its core expertise towards structuring the European Research Area. In this respect, BBMRI-ERIC’s federated access and analysis platform has become a strong asset and reference point for EU initiatives such as the European Health Data Space (EHDS) and EUropean Federation for CAncer IMages (EUCAIM) and serves as an example of the impact of BBMRI-ERIC core expertise on EU health and life science research developments.

**Figure 1.**
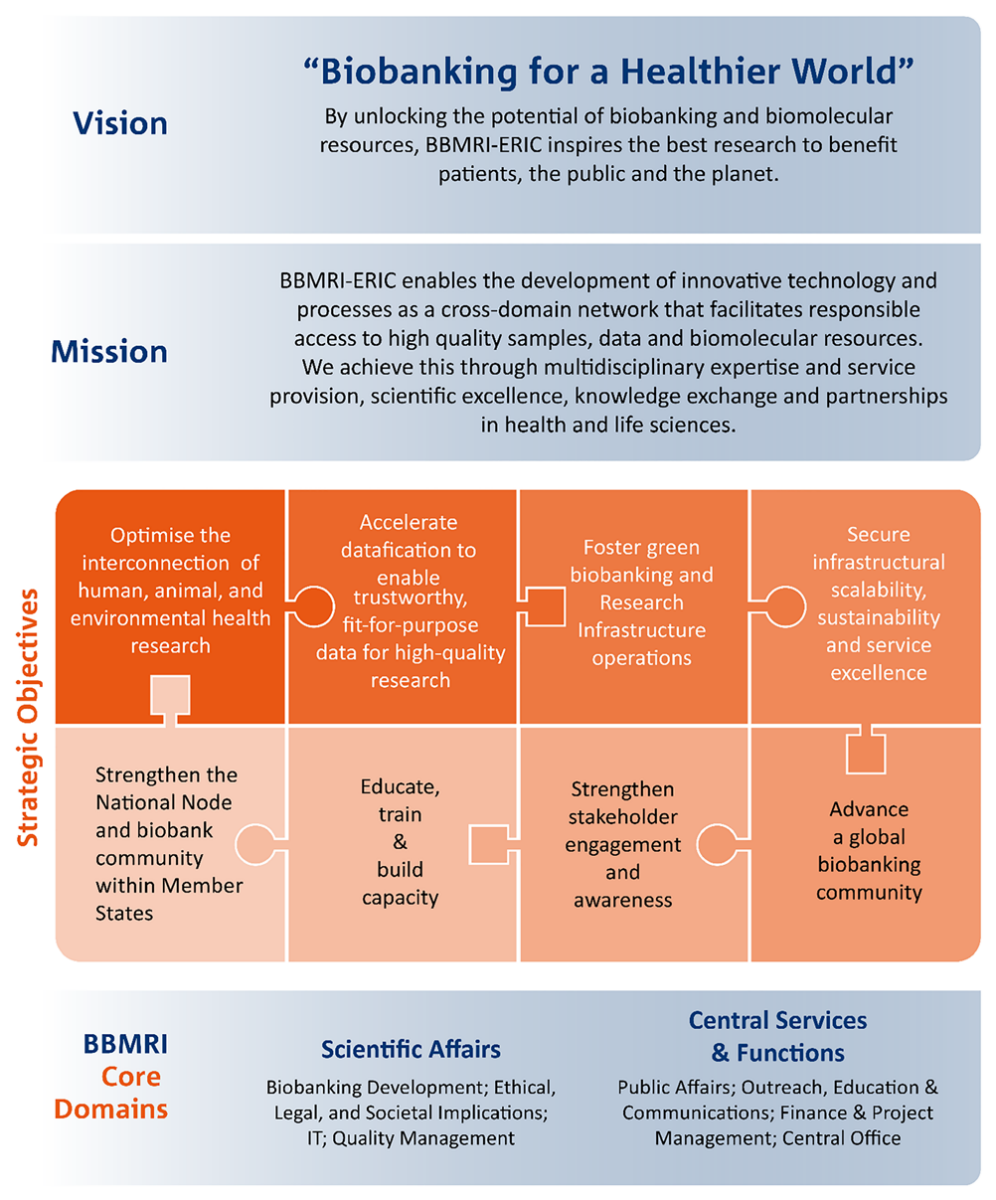
BBMRI-ERIC's 10-Year Roadmap overview.

BBMRI-ERIC’s new 10-Year Roadmap, including its vision, mission and strategic objectives, was outlined, emphasising a One Health approach in biobanking ( Figure 1). This One Health concept, driven by WHO, is an integrated unifying approach recognising that the health of humans, animals, plants, and the wider environment are closely linked and inter-dependent.

Dr Ortega-Paino’s keynote “Sharing is caring: the case of BBMRI Spain”, outlined Spanish research infrastructure and Spain’s involvement in European research infrastructure programmes. Underscoring Spain’s strategic positioning in research infrastructure and its commitment to advancing biobanking through collaboration, quality assurance and innovation, Spain joined BBMRI-ERIC as an observer in 2021, with active participation in working groups and task forces. BBMRI-ERIC observer status is granted for a maximum period of three years, after which Spain will request full membership from the BBMRI-ERIC Assembly of Members. Dr Ortega-Paino reported the benefits of BBMRI-ERIC membership for Spain’s research landscape, including improved data quality, ethical compliance, and streamlined access to research samples and infrastructure. This membership supported enhanced collaboration and integration with EU research networks.

Opening a panel discussion on "Future of Irish Biobanking Community - Roadmap towards BBMRI-ERIC Membership", Dr Sonja Khan, Head of Education at the Institute for Clinical Trials, University of Galway, presented a survey of biobanking practice in Ireland. The study identified 36 biobanks, mostly in Dublin, followed by Galway and Cork, with the majority linked to academic or non-profit institutions and focused on disease-specific research, particularly cancer. The study highlighted infrastructural gaps in the Irish biobanking community, in particular the lack of a national biobanking registry and concluded that a comprehensive biobank registry would enhance transparency and inform government funding ^3^. These findings align with the Health Research Charities Ireland (HRCI) November 2023 report recommending a thorough review of Ireland's biobanking landscape ^4^.

A discussion, exploring Ireland’s potential membership of BBMRI-ERIC followed. Panellists included Mr Billy McCann, a Patient and Public Involvement (PPI) representative and IPPOSI graduate; Dr Avril Kennan, HRCI, Prof Fai Ng, HRB Clinical Research Facility (CRF) UCC, Orlaith Gavan, Pfizer Healthcare Ireland, Dr Robert O’Connor, National Clinical Trials Office (NCTO) Dr Eva Ortega-Paino, Spain and Prof Jens Habermann, BBMRI-ERIC (see Table 1 for full details of speaker affiliations). The discussion was hosted by Emma Snapes, BioConsulting, Cork.

## Methods

Discussions and outcomes from the panel were captured and reproduced with consent from the participants and thematic analysis was conducted to identify key themes related to the benefits, gaps, and strategic imperatives for enhancing Ireland’s biobanking framework. Panel participants and speakers provided consent for their contributions to be included in the study analysis.

**Figure 2.**
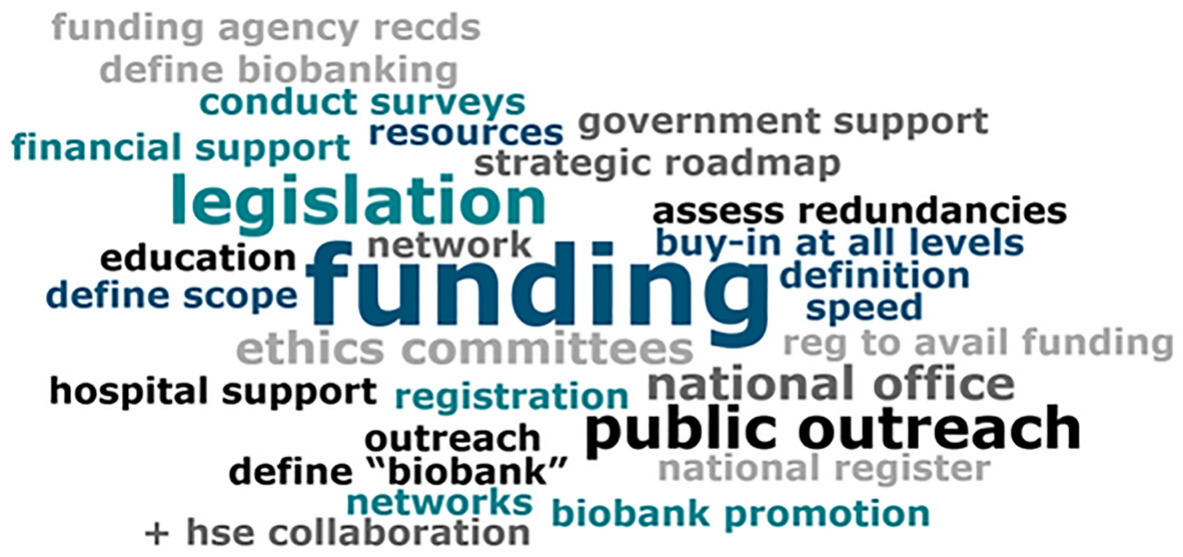
Word cloud of responses to 'What steps can be taken to better map the Irish Biobanking landscape?'

Questions for the panel were prepared by the BioBANC na Gaillimhe organising committee ahead of the symposium, to guide discussions towards achieving strategic goals. During the discussion, VEVOX live polling was used to gauge audience views and questions on posed topics, enabling interactive audience participation and capturing real-time feedback to enrich the discussion. Responses to the question "What steps can be taken to better map the Irish Biobanking landscape?" were visually summarised in a word cloud ( Figure 2), highlighting key themes such as legislation, funding, and public outreach as important in shaping the future of Irish biobanking.

## Results

### Question 1: What steps can be taken to better map the Irish Biobanking landscape?

Dr Eva Ortega-Paino, Secretary General of Spain’s Ministry of Science, Innovation, and Universities, set the stage for the discussion by emphasising the need for foundational education across all stakeholder groups. She argued that education is the starting point for every successful biobanking initiative. According to Dr Ortega-Paino, educating stakeholders as well as the public, is essential for fostering understanding and unity around Ireland’s biobanking goals.

Dr Ortega-Paino discussed Spain’s experience in advancing its biobanking capabilities and how it overcame challenges similar to those faced by Ireland by implementing a national education initiative. This initiative successfully aligned the stakeholders with the biobanking mission, building a supportive and active biobanking community across Spain.

Prof Jens Habermann, Director General of BBMRI-ERIC, emphasised the importance of an inclusive definition of "biobank" in fostering collaboration among patients, scientists, clinicians, and industry representatives. He commended the Irish Health Research Forum’s efforts to unite stakeholders and establish a shared vision, describing it as a significant milestone for Irish biobanking. According to Prof Habermann, a unified vision is key: engaging all stakeholders will set a foundation to propel Irish biobanking forward with strength and cohesion.

This call for stakeholder engagement was echoed by Mr Billy McCann, a PPI Member of the National Biobanking Working Group (NBWG) and the National Research Ethics Committees (NREC) for Medical Devices and an IPPOSI graduate, who spoke about the limited awareness among patients and the public regarding biobanking and its relevance. He recounted his experience learning about biobanking and noted that his peers, although informed about medical research, had little understanding of biobanking’s value. This reflects the need for a more robust public education campaign: “I think a huge job of education needs to be done in the context of the general public.” People want to contribute to research, but they need to better understand how their contributions make a difference. This was further echoed at the National Biobanking Symposium organised by the Biobank Ireland Trust on October 26th 2024.

One of the primary discussions centred around the need to better define and map the biobanking landscape in Ireland. It was emphasised that a comprehensive understanding of what constitutes a biobank and where existing resources are located is crucial. This includes recognising the potential of collections that may not traditionally be classified as biobanks, such as those in hospitals, and the importance of educating both the biobanking community and the broader public about these resources.

Another significant topic was the alignment with international standards and the potential benefits of increased collaboration. The discussions pointed to the importance of creating flexible yet definitive guidelines that could accommodate a wide range of stakeholders, including patient representatives, clinicians, researchers, and industry professionals. Establishing clear roadmaps and fostering cooperative initiatives were seen as essential steps towards developing a coherent strategy for biobanking in Ireland.

Funding and sustainability also emerged as critical themes. The need for more robust funding mechanisms was highlighted, with suggestions that biobanking should be integrated into the healthcare system's infrastructure, akin to essential diagnostic tools. This would ensure long-term sustainability and efficacy, moving beyond the limitations of short-term research grants.

### Question 2: What are the benefits of joining BBMRI-ERIC—why is it important?

The discussion on the benefits of Ireland joining BBMRI-ERIC highlighted the strategic and collaborative advantages of such a step. The dialogue emphasised the necessity of transcending isolated efforts in biobanking within Ireland and leveraging a broader European network.

Prof Jens Habermann emphasised the need for a collaborative approach in biobanking, stating, “It's a very dynamic field, and you need to involve all stakeholders, including patient representatives, scientists, clinicians, industry, and the public.” He strongly advocated against operational isolation by highlighting, “we cannot continue working in silos.” Prof Habermann argued that joining BBMRI-ERIC would seamlessly integrate Ireland into a global network characterised by standardised, regulated, and collaborative practices. Such membership would not only unlock the full potential of Ireland’s biobanks but also significantly support both public and private sectors in building a healthcare system equipped to address the nation’s complex health challenges.

The discussions also touched upon the need for standardisation and efficiency in biobanking processes, which could be significantly enhanced through Ireland’s membership of BBMRI-ERIC. This would not only facilitate improved research outcomes but also ensure that Irish biobanking practices are aligned with international best practices and regulatory standards.

Panellists discussed the dual benefits of joining BBMRI-ERIC from both a strategic and ethical standpoint. By integrating into this network, Ireland would not only improve its biobanking infrastructure but also contribute to and benefit from collective European efforts in medical research and healthcare advancements. This cooperation is seen as crucial for facing the rapid pace of change in global healthcare needs and research demands.

Dr Robert O'Connor added a critical perspective on the operational challenges and regulatory aspects of biobanking. He argued for the inclusion of Ireland in BBMRI-ERIC from an infrastructural and economic perspective. He mentioned that as European citizens, Irish researchers and institutions have much to gain from and contribute to this collective endeavour. He discussed the importance of standardisation, using his experiences to illustrate how variations in medical testing across hospitals could affect patient care more significantly than the conditions being tested.

### Question 3: What gaps need to be addressed to join, and how should they be addressed?

In addressing the third question posed to the panel about the steps Ireland needs to take to join BBMRI-ERIC, the discussion largely highlighted Ireland's readiness and strong potential for aligning with European biobanking standards.

Prof Jens Habermann, Director General of BBMRI-ERIC, underscored that no significant barriers exist to delay Ireland’s inclusion, advocating for swift action: “There is no gap that needs to be overcome first. Join - we will do the rest together. Ireland has all it takes to become a member.”

Other panellists pointed out specific areas needing attention, such as the lack of a comprehensive ethical framework specific to biobanking in Ireland. This gap is seen as a barrier to standardising biobanking practices to meet international norms. Another highlighted gap was the perceived lack of political awareness and will. This absence of biobanking on the political agenda could suggest politicians are not pressured to act because they are not informed about the importance of biobanking. The Secretary General for Research Ministry of Science Innovation and Universities of Spain, Dr Eva Ortega-Paino, echoed this sentiment stressing the need for proactive engagement with politicians to raise awareness and foster legislative support.

The fragmentation of the Irish biobanking community has been recognised as a significant obstacle to realising the full potential of biobanking as a critical enabler of research. This fragmentation hinders the HRB’s ability to appreciate the integral role biobanking plays in the research they fund. Addressing this gap aligns with Objective 4 of the HRB Strategy 2021–2025, which emphasises the creation of a thriving research environment ^5^.

### Question 4: Are there any parallels with clinical trials—European Clinical Research Infrastructure Network (ECRIN)? If so, what can the biobanking community learn from clinical trials and vice versa?

The panel discussion delved into the parallels between clinical trials network ECRIN ^6^ and biobanking, particularly focusing on what the biobanking community could learn from the success and integration of clinical trials into a European framework.

In 2022, the European Clinical Research Infrastructure Network (ECRIN) partnered with the European Research Infrastructure for Biobanking and Biomolecular Resources (BBMRI-ERIC) and the European Infrastructure for Translational Medicine (EATRIS-ERIC) to establish the European Alliance of Medical Research Infrastructures (EU-AMRI) ^7^. This alliance was formed to deliver complementary services to researchers in the field of biomedical sciences, with a focus on advancing personalised medicine and the development of new therapeutic approaches. The EU-AMRI was officially launched on April 5, 2022, in Brussels. During the launch event, the directors of the three infrastructures outlined the strategic goals of the alliance and the significance of collaborative efforts in expediting patient access to innovative treatments ^8^.

Dr Robert O'Connor/and other panellists discussed the parallels between ECRIN and BBMRI-ERIC, considering the former's 13-member state membership and how Ireland’s HRB provides funding through the Department of Health (DoH) for ECRIN membership. He noted the different nature of BBMRI-ERIC, which focuses more on biobanking compared to ECRIN’s investigator-led trials. Dr Robert O’Connor stressed the importance of not waiting for perfect conditions to join BBMRI-ERIC, advocating for concurrent actions in addressing legislative gaps and improving biobanking processes to maximise the benefits of membership.

Similarly, Ms Oonagh Ward highlighted the similarities in complexity between the ECRIN and BBMRI-ERIC landscapes but pointed out that ECRIN had a strategic plan and strong government support that facilitated its success. She emphasised the need for a similar strategic approach for biobanking in Ireland to effectively join and benefit from BBMRI-ERIC.

The importance of standardisation across biobanking was underscored, noting that aligning with European standards can significantly enhance credibility and public trust in biobanking practices. Such standardisation not only assures patients donating samples for research of the integrity and safety of the processes but also paves the way for international collaborations that are vital for advancing personalised medicine.

Orlaith Gavan, Country Medical Director at Pfizer Healthcare Ireland, emphasised the pharmaceutical sector's strong backing for advancing biobanking infrastructure. Noting that the shift toward personalised treatments depends on robust biobanking systems, which enable tailored therapeutic development, Orlaith Gavan also pointed out that a well-supported biobanking infrastructure could significantly boost Ireland's competitiveness in attracting novel clinical trials, a key aspect of drug development and evidence-based healthcare. She underscored biobanking’s vital role in pharmaceutical R&D, positioning it as a cornerstone for driving innovation in medical solutions.

Prof Fai Ng, Director at the HRB CRF UCC, shared valuable insights into the structure and funding of biobanking. Drawing on his UK experience, he proposed strategies to strengthen biobanking initiatives in Ireland. He emphasised the importance of national and regional support, suggesting that Research Ireland (formerly Science Foundation Ireland) could adopt a role akin to that of the UK’s Medical Research Council in advancing biobanking infrastructure. Prof Ng also emphasised the need to establish a national registry for biobanks, facilitating streamlining access and increased utilisation, thereby enhancing their value as a shared resource. His recommendations underscore the importance of adapting successful international strategies to bolster Irish biobanking for both national and global research efforts.

## Discussion

The panel emphasised the need for a strategic approach to overcome legislative and procedural challenges in Irish biobanking. Aligning with European standards and pursuing BBMRI-ERIC membership were discussed as key steps. These measures would enhance research capabilities and enable more effective, personalised healthcare solutions.

A vision for a diverse and inclusive Irish biobanking network was also highlighted, stressing the importance of representing all population segments to ensure comprehensive and impactful health research in a multicultural society. The panel advocated for immediate and proactive steps towards integrating into the European biobanking framework through BBMRI-ERIC. This approach is a vital step toward standardisation, collaboration, and advancing Irish healthcare, representing a commitment to patients, researchers, and industry partners.

**Figure 3.**
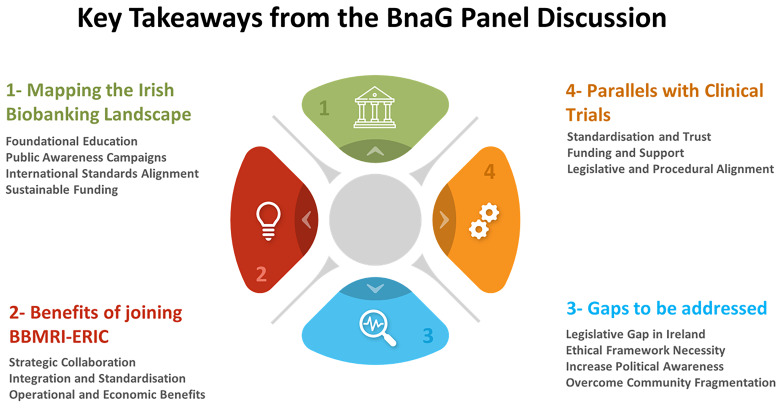
Key takeaways from the BnaG panel discussion.

The panel’s consensus was clear: Ireland’s healthcare and research infrastructures would benefit greatly from the integration, standardisation, and collaborative opportunities that BBMRI-ERIC membership provides. For Ireland, joining this network is not merely an opportunity; it is an obligation to the patients, researchers, and industry partners who are committed to advancing Irish healthcare. Joining BBMRI-ERIC is a decisive step toward fulfilling this commitment, and Ireland is poised to make that leap, equipped with the support of its dedicated stakeholders and a unified vision ( Figure 3).

Audience polling revealed strong support for Ireland joining BBMRI-ERIC, with 100% of respondents in favour and 75% willing to provide contact details for further engagement. Stakeholders’ primary interest lay in biobanking’s future, with 60% from academia, 14% from government agencies, and 7% each from hospitals and charitable organisations. This highlights the interdisciplinary nature of biobanking, uniting healthcare, regulatory, and non-profit sectors. The unanimous support underscores the community’s commitment to strengthening Ireland’s biobanking infrastructure and its international research role.

This engaging exchange reinforced a shared commitment among stakeholders to strengthen Ireland’s biobanking capabilities and prioritise membership in BBMRI-ERIC, a pivotal step in fostering international collaboration and advancing healthcare outcomes. Joining BBMRI-ERIC is seen as essential for aligning Ireland’s biobanking infrastructure with global standards, improving patient care, and unlocking broader research and economic opportunities.

The session on "Quality Matters" highlighted the importance of standards and best practices in biobanking. Linda Hendy from the National Standards Authority of Ireland (NSAI) and William Clarke from the Irish National Accreditation Board (INAB) emphasised the critical roles of standardisation, accreditation, and compliance in ensuring the credibility of biobanking operations and fostering international collaboration. Dr Nicola Miller and Dr Oliver Carroll (University of Galway) outlined International Society for Biological and Environmental Repositories (ISBER) Best Practices and the value of proficiency testing in maintaining material fitness and enhancing performance. Jack O'Grady (My Green Lab) discussed promoting environmental sustainability in laboratory operations.

Dr Richard Flavin (Trinity College Dublin) showcased Biobank Ireland Trust’s efforts in providing high-quality tissue samples for cancer research and fostering a federated biobank network, advocating for patient and public involvement (PPI) and ethical governance. Prof Seán O. Hynes (University of Galway/University Hospital Galway) concluded the session by reaffirming the commitment to advancing research, improving healthcare outcomes, and solidifying Ireland’s role in the global biobanking landscape.

## Conclusion

BioBANC Symposium III demonstrated the strong support for networked biobanking and BBMRI-ERIC membership from the stakeholders in Ireland’s biobanking community, patient advocacy groups, and the pharmaceutical industry. The infrastructural support is crucial for strengthening the foundation of biobanking in Ireland and enhancing its impact on global biobanking practices. Furthermore, there is a critical need to address human capital gaps to facilitate the activation of BBMRI-ERIC membership and ensure preparedness upon approval.

Membership in BBMRI-ERIC would align Ireland with leading European research infrastructures, enhancing its research capabilities and fostering international collaborations. It would also promote higher standards and economic growth through improved research infrastructure. The discussions and strategies outlined at the symposium provide a roadmap for advancing Ireland’s biobanking capabilities to effectively meet current and future health research challenges.

“ *BioBANC Symposium has become an essential platform for bringing together the specialised community of Irish biobanking professionals. As biobanks continue to evolve, they are playing an increasingly critical role in advancing healthcare and addressing some of the world’s most pressing health challenges. This evolution is bringing changes in the relationship between biobanks and the biological samples and data they are entrusted to preserve by patients and participants. The Irish biobanking community is advocating that these changes are best navigated in alignment with our European partners through membership of BBMRI-ERIC. Láidir le chéile”.*

Dr Nicola Miller, Dr Sonja Khan – BioBANC na Gaillimhe

*“BBMRI-ERIC membership significantly enhances member states’ health research capabilities by providing access to resources of almost 500 biobanks, while ensuring adherence to biobanking standards and ethical, legal and societal aspects. Ireland's membership would align with the BBMRI-ERIC 10-Year Roadmap "Biobanking for a Healthier World," which aims to unlock the potential of biobanking and biomolecular resources to inspire the best research for the benefit of patients, the public, and the planet. We therefore hope for Ireland to join our community.”*

Prof Jens K. Habermann – Director General, BBMRI-ERIC

*“A huge job of education needs to be done in the context of the general public. People want to contribute to research, but they need to better understand how their contributions make a difference”*

Mr Billy McCann – PPI Member NBWG, NREC for Medical Devices, IPPOSI graduate.

*"We simply owe it to our patients. We are not doing anywhere close to enough. For patients, when they attend our hospital and social care services, and if research is embedded, that could be completely different. Let's just get on with it."*

Dr Avril Kennan – CEO Health Research Charities Ireland (HRCI)

*“Pfizer invests a significant amount in Ireland and recognises the importance of biobanking, which is why it is built into all clinical trials. To maintain Ireland’s position in oncology, we simply must improve, if we are to continue to position Ireland as world class”.*

Orlaith Gavan – Chief Medical Officer, Pfizer Healthcare, Ireland

*"We need to involve all stakeholders - patient representatives, scientists, clinicians, industry, and the public - to leverage existing expertise, tools, and innovation, creating new synergies. We must move beyond working in silos to fully leverage resources and expertise. This is crucial as we face dramatic global challenges; we cannot afford to waste these resources."*

Prof Jens K. Habermann – Director General, BBMRI-ERIC

*“We in University of Galway are fortunate to have our translational research facility and biobank co-located with the main teaching hospital and cancer centre in the West and Northwest of Ireland. As Director of the regional Cancer Network, biobanks and multidisciplinary teams working across hospital and academia are essential to enabling patient focused research. I am delighted to see this BioBANC Symposium taking place, encouraging collaboration and knowledge exchange between researchers, clinicians, educators, and members of the public.”*

Prof Michael Kerin – Chair of Surgery, University of Galway (formerly NUI Galway), Director of the HSE West and North West Cancer Network and Vice President of the Royal College of Surgeons in Ireland.

## Ethics and consent

The manuscript reports on a panel discussion held during the BioBANC Symposium, which featured invited experts discussing publicly available information and their professional opinions. As this was a symposium rather than a study involving the systematic collection of data from human subjects, no formal ethical approval was required. The format of the panel discussion did not involve interventions with or treatments of human subjects as defined under the Declaration of Helsinki, nor did it include any personal or sensitive data subject to ethical oversight.

Consent for including the names and contributions of the panellists was obtained. All panellists were contacted via email and agreed to have their discussions and professional opinions included in the report.

## Data Availability

Data from VEVOX polling conducted during the BioBANC Symposium, includes aggregated stakeholder opinions on biobanking and BBMRI-ERIC membership, is used in the discussion to contextualise community engagement and support. To ensure participant privacy and comply with data protection standards, the raw polling data is not publicly accessible. However, summary data and key insights are incorporated directly into the manuscript text. Additionally, a word cloud ( Figure 2) generated from VEVOX polling results is included to visually represent the support for enhancing Ireland's biobanking infrastructure.
