# Supplementary material for: A roadmap towards Ireland’s membership of BBMRI-ERIC
Source: HRB Open Res. 2025 May 29;8:50. Originally published 2025 Apr 4. [Version 2] doi: 10.12688/hrbopenres.14089.2 (PMC12038341; doi:10.12688/hrbopenres.14089.2)
Supplement: Peer review history

# Reviewer report: Reviewer response for version 2

**DOI:** 10.21956/hrbopenres.15585.r47511

**Published:** 2025-06-16

**Authors:** Daniela Capello

**Affiliations:** 1 Department of Translational Medicine, University of Eastern Piedmont, Novara, Italy

Approved. No further comments. Congratulations on the work done

Is the work clearly and accurately presented and does it cite the current literature?

Yes

If applicable, is the statistical analysis and its interpretation appropriate?

Not applicable

Are all the source data underlying the results available to ensure full reproducibility?

No source data required

Is the study design appropriate and is the work technically sound?

Yes

Are the conclusions drawn adequately supported by the results?

Yes

Are sufficient details of methods and analysis provided to allow replication by others?

Yes

Reviewer Expertise:

Biobanking quality and sustainability, technical and ELSI aspects, public and patient engagement, population-based studies, biomarker discovery

I confirm that I have read this submission and believe that I have an appropriate level of expertise to confirm that it is of an acceptable scientific standard.

# Reviewer report: Reviewer response for version 2

**DOI:** 10.21956/hrbopenres.15585.r47510

**Published:** 2025-06-03

**Authors:** Zisis Kozlakidis

**Affiliations:** 1 International Agency for Research on Cancer, Rhone Alpes, France

No further comments.

Is the work clearly and accurately presented and does it cite the current literature?

Yes

If applicable, is the statistical analysis and its interpretation appropriate?

Not applicable

Are all the source data underlying the results available to ensure full reproducibility?

Yes

Is the study design appropriate and is the work technically sound?

Yes

Are the conclusions drawn adequately supported by the results?

Yes

Are sufficient details of methods and analysis provided to allow replication by others?

Yes

Reviewer Expertise:

Laboratory infrastructure, biobanking, biobanking science, digital health.

I confirm that I have read this submission and believe that I have an appropriate level of expertise to confirm that it is of an acceptable scientific standard.

# Reviewer report: Reviewer response for version 1

**DOI:** 10.21956/hrbopenres.15478.r46682

**Published:** 2025-04-30

**Authors:** Andrea Wutte

**Affiliations:** 1 BBMRI-ERIC, Korneuburg, Austria

Notes to the authors:

 The manuscript is well written and gives the reader an good overview on the discussion during the BioBANC Symposium.  

 Notes to: 

**1. Is the work clearly and accurately presented and does it cite the current literature?**

- *Reference #1* directs to a general EU Commission page on research infrastructures, while the text specifically mentions BBMRI-ERIC. This should be clarified, and a direct link to the BBMRI-ERIC website would be helpful.
- *Reference #3* cites a dissertation referenced by Dr. Sonja Kahn. Is this dissertation publicly accessible? If so, please provide a direct link.
- *References #2 and #5* point to the same source—are there any differences between them? If not, consider consolidating.
- *Reference #7* should link to EU-AMRI, as per the text. The correct link is: https://eu-amri.org.

Note to

**2. If applicable, is the statistical analysis and its interpretation appropriate?**

 In the discussion, the authors should provide reference figures (e.g., total number of audience polling = 100%) to clarify what the presented percentages represent.

 To reinforce the point raised in Question 1—“better define and map the biobanking landscape in Ireland”—the authors could explain how the audience’s affiliations (e.g., academia, hospitals) reflect the broader national landscape. Including national-level benchmarks would help contextualize the data in a bigger picture. For example, how representative is the symposium audience of the wider group of stakeholders who could benefit from initiatives like BioBANC hence would benefit from joining BBMRI-ERIC?

Is the work clearly and accurately presented and does it cite the current literature?

Partly

If applicable, is the statistical analysis and its interpretation appropriate?

Partly

Are all the source data underlying the results available to ensure full reproducibility?

Yes

Is the study design appropriate and is the work technically sound?

Yes

Are the conclusions drawn adequately supported by the results?

Yes

Are sufficient details of methods and analysis provided to allow replication by others?

Yes

Reviewer Expertise:

Biologist, certified Quality Manager, certified Lead auditor and technical assessor for Accreditation bodies (eg. Biobanking and Pre-analytical standards) . 10 y Experience in Biobanking  and building a European biobank network.

I confirm that I have read this submission and believe that I have an appropriate level of expertise to confirm that it is of an acceptable scientific standard, however I have significant reservations, as outlined above.

# Reviewer report: Reviewer response for version 1

**DOI:** 10.21956/hrbopenres.15478.r46679

**Published:** 2025-04-28

**Authors:** Daniela Capello

**Affiliations:** 1 Department of Translational Medicine, University of Eastern Piedmont, Novara, Italy

Overall, this is a well-written paper that highlights the scientific, ethical, and societal benefits of increasing awareness about biobanking and the opportunities that BBMRI-ERIC membership offers to enhance the quality and coherence of national efforts. In particular, the article underlines the potential for growth in scientific excellence, ethical governance, and community engagement. At the same time, it thoughtfully addresses key challenges, such as the limited political awareness, the fragmentation of the current biobanking landscape, and the importance of fostering greater public and patient understanding of their essential role in advancing research, primarily through biobanking.

 These considerations are relevant not only for countries like Ireland, which is in the process of joining BBMRI-ERIC, but also for member countries, which must continue to nurture awareness and uphold best practices to maximize the value of biobanking infrastructures.

 Here are a few suggestions for improvement:

 -  The manuscript refers throughout to the *“BioBANC Symposium”*, but the term *BioBANC* itself is not clearly defined. It would be helpful to explain early in the text what *BioBANC* stands for—whether it is a formal initiative, a project, or a recurring event—and to clarify its role within the Irish biobanking community.

 - The abbreviation *BnaG* is listed among the abbreviations, but it is not used anywhere in the body of the manuscript.

 - The acronyms *HSE* and *IPPOSI* are used in the text but are not spelled out on first use, nor are they listed among the abbreviations. For clarity, particularly for international readers, please consider spelling out *HSE* and *IPPOSI* when they are first mentioned and including them in the list of acronyms.

 - VEVOX polling is mentioned multiple times and appears to have provided valuable real-time feedback during the symposium. However, the manuscript would benefit from a brief explanation of what VEVOX is, particularly for readers unfamiliar with the tool. It would also be helpful to include additional methodological details, for example, the number and type of questions posed (e.g., multiple-choice, free-text), the overall number of participants who responded, and how the responses were analyzed or summarized. This would provide helpful context for interpreting the visual elements (e.g., the word cloud) and the polling insights discussed in the results and discussion sections.

 Additional suggestions for improvement:

 The "Quality Matters" session in the Discussion section is briefly summarised through a list of names and contributions, but it would benefit from better contextualisation. Clarifying the objectives of this session, its relation to the overall symposium theme, and how it connects to the roadmap towards BBMRI-ERIC membership would help the reader better understand its relevance and impact.

 The brief reference to the Biobank Ireland Trust could also be more clearly linked to the rest of the article. Specifically, it would be helpful to clarify Biobank Ireland Trust's relationship to BioBANC na Gaillimhe or the national biobanking framework and whether it is part of the same strategic network or represents a complementary initiative. This would provide readers with a more cohesive understanding of the national landscape.

Is the work clearly and accurately presented and does it cite the current literature?

Yes

If applicable, is the statistical analysis and its interpretation appropriate?

Not applicable

Are all the source data underlying the results available to ensure full reproducibility?

No source data required

Is the study design appropriate and is the work technically sound?

Yes

Are the conclusions drawn adequately supported by the results?

Yes

Are sufficient details of methods and analysis provided to allow replication by others?

Yes

Reviewer Expertise:

Biobanking quality and sustainability, technical and ELSI aspects, public and patient engagement, population-based studies, biomarker discovery

I confirm that I have read this submission and believe that I have an appropriate level of expertise to confirm that it is of an acceptable scientific standard, however I have significant reservations, as outlined above.

# Response

**Published:** 2025-05-22

**Authors:** Amina Arar

**Affiliations:** University of Galway, Galway, County Galway, Ireland

We thank the reviewer for their thoughtful and encouraging feedback. We appreciate the valuable suggestions provided to improve clarity and coherence. We have addressed each of the points below:

**R1 and R2.** To provide greater clarity, we have added a sentence early in the Introduction explaining that BioBANC na Gaillimhe is a multidisciplinary initiative based at the School of Medicine, University of Galway, which brings together expertise in biobanking with the aim of building an inclusive, patient-centered, and efficient research environment. The term “BioBANC” refers to the initiative, while the annual BioBANC Symposium is one of its key national engagement activities.

 Regarding the BnaG, it stands for BioBANC na Gaillimhe, the Irish term loosely translates to “Galway Biobank.” It is a locally used abbreviation and has been included in Figure 3 the visual summary of key recommendations from the panel discussion.

**R3.** HSE (Health Service Executive) and IPPOSI (Irish Platform for Patient Organisations, Science and Industry) were spelled out at first mention and added both to the list of abbreviations.

**R4. **VEVOX is an interactive live polling and audience engagement tool that was used during the panel discussion to enhance inclusivity and encourage real-time participation. A QR code linking to the VEVOX session was circulated by email to attendees in advance  of the event (available at: https://stories.universityofgalway.ie/biobanc-na-gaillimhe-bnag3/index.html).

 Participants were able to respond via their devices, using both multiple-choice and free-text formats. The polling enabled instant feedback and inclusive engagement, with free-text responses automatically visualized as a word cloud to support discussion.

**R5.** The “Quality Matters” session was indeed rich in content and aligned closely with the overarching goals of the symposium. To better contextualise its relevance, we have added a passage to the manuscript summarising the key presentations and their connection to biobanking standards and international collaboration. While the main focus of the paper remains the panel discussion and Ireland’s potential membership in BBMRI-ERIC, we agree that this session adds important depth to the national conversation around quality and best practices, and we appreciate the opportunity to clarify its contribution.

**R6. **We thank the reviewer for this suggestion. The relevant section has now been revised to clarify that Biobank Ireland Trust, presented by Professor Richard Flavin (Trinity College/St James’s Hospital, Dublin), is an independent national charitable initiative that supports the development of biobanking throughout Ireland, with a particular focus on cancer research. It promotes collaboration through a federated model and encourages best practices in biobanking. Biobank Ireland Trust is also a member of the Mapping Ireland Biobanking Working Group and contributes to national discussions around biobanking infrastructure, including those highlighted at the BioBANC Symposium.

# Reviewer report: Reviewer response for version 1

**DOI:** 10.21956/hrbopenres.15478.r46677

**Published:** 2025-04-27

**Authors:** Zisis Kozlakidis

**Affiliations:** 1 International Agency for Research on Cancer, Rhone Alpes, France

The article provides a well-structured summary of a panel discussion on biobanking that took place during the BIOBANC conference in Ireland, discussing Ireland's possibility of joining BBMRI-ERIC.

 - The experience with ECRIN is mentioned in the article, however, it is not clear if this is the only ERIC that Ireland is a member of. It would be good to understand the extend to which Ireland participates to ERICs in general.

 - It would be good if Questions 1 and 4 could be shortened slightly.

 - In the discussion, it is not surprising that 100% of participants in a dedicated thematic conference are interested in the further development of that field. While it demonstrates clear interest, it should be counterbalanced in the knowledge that not all Irish biobanks/biobankers were represented.

 - Please provide the reference for the ISBER Best Practices and proficiency testing.

 - The discussion mentions "...panel advocated for immediate and proactive steps...", but no specific example is provided.

Is the work clearly and accurately presented and does it cite the current literature?

Yes

If applicable, is the statistical analysis and its interpretation appropriate?

Not applicable

Are all the source data underlying the results available to ensure full reproducibility?

Yes

Is the study design appropriate and is the work technically sound?

Yes

Are the conclusions drawn adequately supported by the results?

Yes

Are sufficient details of methods and analysis provided to allow replication by others?

Yes

Reviewer Expertise:

Laboratory infrastructure, biobanking, biobanking science, digital health.

I confirm that I have read this submission and believe that I have an appropriate level of expertise to confirm that it is of an acceptable scientific standard, however I have significant reservations, as outlined above.

# Reviewer report: Reviewer response for version 1

**DOI:** 10.21956/hrbopenres.15478.r46683

**Published:** 2025-04-18

**Authors:** Mieke Dewilde

**Affiliations:** 1 Belgian Cancer Registry, Brussels, Belgium

In general, this is an important and clear article.

 I have some minor suggestions.

 In Introduction, alinea 3:

 ...through membership of the European Clinical Research Infrastructure Network (ECRIN) the European research infrastructure (ERIC) ...:

 this reads a bit difficult for a foreign speaker. Maybe add ..through membership of ...(ECRIN)... in the ... (ERIC)...,

 or: ... Through membership of ... the European Research Infrastructure Consortium (ERIC) - European Clinical Research Infrastructure Network (ECRIN).

 In Introduction, alinea 5:

 ..., ERIC Forum, which BBMRI-ERIC coordinates...

 I read this sentence as 'BBMRI-ERIC is coordinating the ERIC Forum', and I'm unsure if this is what is meant, when I look at ERIC Forum Executive Board -.

 Methods:

 The methodology used and thoroughness of the discussion is impressive, and the methods of capturing and recording the voices of the stakeholders are both effective and refreshing.

 Results, question 1, alinea 4:

 It would benefit clarity is PPI is first spelled out here instead of in the last alinea of the discussion.

 What does the acronym IPPOSI stand for?

 Discussion

 The discussion is thorough and nails the point. I'm delighted to read about the strong support to further promote networked biobanking and pursue BBMRI-ERIC membership. 

 Alinea 6:

 .. ISBER Best Practices...: to ensure reader refer to the correct edition, it might be beneficial to include a reference to Snapes E, et al., 2023 (Ref 1) or ISBER Best Practices For Repositories - ISBER.

Is the work clearly and accurately presented and does it cite the current literature?

Yes

If applicable, is the statistical analysis and its interpretation appropriate?

Not applicable

Are all the source data underlying the results available to ensure full reproducibility?

No source data required

Is the study design appropriate and is the work technically sound?

Yes

Are the conclusions drawn adequately supported by the results?

Yes

Are sufficient details of methods and analysis provided to allow replication by others?

Yes

Reviewer Expertise:

Biobanking operations, Biobanking quality management, BBMRI-ERIC membership as national node personnel, Biobanking norms and standards.

I confirm that I have read this submission and believe that I have an appropriate level of expertise to confirm that it is of an acceptable scientific standard.

## References

[1] : Updating International Society for Biological and Environmental Repositories Best Practices, Fifth Edition: A New Process for Relevance in an Evolving Landscape. *Biopreserv Biobank* .2023;21(6) : 10.1089/bio.2023.0140 537-546 10.1089/bio.2023.0140 38149936

# Response

**Published:** 2025-05-22

**Authors:** Amina Arar

**Affiliations:** University of Galway, Galway, County Galway, Ireland

We thank the reviewer for their positive feedback and helpful suggestions. We have addressed all the points raised, as outlined below:

 R1. Introduction, Alinea 3:

 The sentence has now been revised for clarity. It now reads: “…through membership of the European Clinical Research Infrastructure Network (ECRIN) within the European Research Infrastructure Consortium (ERIC)...”

 R2. Introduction, Alinea 5:

 The role of BBMRI-ERIC was clarified by rephrasing to: “ This network includes the ERIC Forum, which is administratively supported by BBMRI-ERIC, and other European Research Infrastructures in the field of health and life sciences.” 

 R3. We thank the reviewer for this positive feedback. No changes were necessary in response to this comment.

 R4. Results – Question 1, Alinea 4:

  “Patient and Public Involvement (PPI)” has been spelt at the first mention and IPPOSI defined as the “Irish Platform for Patient Organisations, Science and Industry.”

 R5. Discussion, Alinea 6:

 The recommended reference to the ISBER Best Practices (Snapes et al., 2023) article and The ISBER Best Practices, Fifth Edition has been added.

 We thank the reviewer once again for their thoughtful review and support of the work.
