# Supplementary material for: A roadmap towards Ireland’s membership of BBMRI-ERIC
Source: HRB Open Res. 2025 Apr 4;8:50. [Version 1] doi: 10.12688/hrbopenres.14089.1 (PMC12038341; doi:10.12688/hrbopenres.14089.1)
Supplement: Peer review history

# Response

**Published:** 2025-05-22

**Authors:** Amina Arar

**Affiliations:** University of Galway, Galway, County Galway, Ireland

We thank the reviewer for their thoughtful feedback and helpful suggestions. We have addressed each point as follows:

**R1**. Regarding Ireland’s participation in other ERICs, in addition to ECRIN-ERIC, Ireland is also a member of several other European Research Infrastructure Consortia. Notably, Ireland is a member of EATRIS-ERIC, which supports translational medicine by bridging the gap between laboratory research and clinical application. Ireland also participates in ERICs such as CESSDA-ERIC (social science data), ESS-ERIC (European Social Survey), EMSO-ERIC (marine observation), Euro-Argo ERIC (ocean profiling), and LOFAR ERIC (radio astronomy). However, we focused on ECRIN in this article as it plays the most relevant role in the context of clinical trials and biobanking, which is the primary subject of the paper.

**R2**. We acknowledge the reviewer’s comment. During the panel discussion, Questions 1 and 4 prompted particularly rich and detailed responses from the participants due to the significance of the topics. Question 1 addressed critical steps needed to map the Irish biobanking landscape, and question 4 explored valuable parallels with the clinical trials network ECRIN. We have aimed to preserve the depth of ideas shared while improving the overall readability.

**R3**.  We thank the reviewer for this thoughtful and important observation. We acknowledge that while the polling results reflect strong interest among BioBANC Symposium attendees, the views captured represent a subset of the broader biobanking community in Ireland. To address this, a sentence has been added to the manuscript noting this limitation and clarifying that not all Irish biobanks or biobankers were present: “It should be noted that polling results reflect the views of participants who attended the symposium and may not represent the entire Irish biobanking landscape.”

**R4**. We thank the reviewer for highlighting the need to include a reference for ISBER Best Practices and the associated proficiency testing program. The citation for the ISBER Best Practices (Snapes et al., 2023) has been added (9,10), along with reference to the ISBER endorsed IBBL proficiency testing scheme (11,12). We also added two sentences to the "Quality Matters" section of the manuscript.

 “The ISBER-endorsed Biospecimen Proficiency Testing Program, provided by the Integrated Biobank of Luxembourg (IBBL) enables biorepositories to benchmark their performance against global peers, meet accreditation standards (e.g., ISO 20387, ISO 17025, CLIA), and continuously monitor and improve testing and processing accuracy (11, 12).”

**R5.** We thank the reviewer for raising this point. In response, we are including a concrete recommendation from the roadmap below. Additionally, Figure 3 has been used to encapsulate the key takeaways from the panel discussion.

 Key Recommendations from the Roadmap:

1. Join BBMRI-ERIC: Position Ireland as a full member of the European biobanking network.
2. Integrate biobanking into the health system: Embed biobanking in national healthcare policies to support ongoing research.
3. Secure infrastructure funding: Move beyond short-term grants to a sustainable funding model.
4. Strengthen public and political understanding: Launch awareness campaigns to enhance trust and transparency.
5. Create a national biobanking node: Coordinate efforts and standardise best practices across the country.

We appreciate the reviewer’s expertise and constructive input, to improve the clarity of the paper.
